# Finerenone as adjunctive therapy for residual proteinuria in lupus nephritis: a case series of 10 patients

**DOI:** 10.3389/fmed.2026.1852929

**Published:** 2026-07-03

**Authors:** Rui Gao, Jianan Wang, Qi Liu, Ming Li

**Affiliations:** 1 School of Clinical Medicine, Shandong Second Medical University, Weifang, Shandong, China; 2 Weifang People's Hospital, Shandong Second Medical University, Weifang, Shandong, China

**Keywords:** finerenone, kidney protection, lupus nephritis, mineralocorticoid receptor antagonists, residual proteinuria

## Abstract

**Introduction:**

After baseline immunosuppression and renin-angiotensin system inhibitor (RASi) treatment for lupus nephritis (LN), the persistence of residual proteinuria is a core risk factor for poor renal prognosis, which is closely related to continuous progression of renal inflammation and fibrosis and long-term elevated risk of end-stage renal disease. Finerenone is a highly selective non-steroidal mineralocorticoid receptor antagonist (MRA) that has been proven to reduce proteinuria and exert renal protective effects in diabetic nephropathy. However, its association with proteinuria changes and safety profile in residual proteinuria of LN remain.

**Methods:**

This single-center retrospective case series enrolled 10 patients with clinically diagnosed LN who had residual proteinuria (24-h urine protein quantification ≥0.5 g) despite ≥6 months of standard treatment. Finerenone 10 mg once daily was added to the original treatment, and patients were followed for 24 weeks. The primary endpoint was change in 24-h urine protein quantification; secondary endpoints included serum albumin, estimated glomerular filtration rate (eGFR), and safety parameters. Data were presented as median (interquartile range, P25, P75). Repeated measurements were compared using the Friedman test with Bonferroni correction.

**Results:**

At baseline, 12 weeks, and 24 weeks, 24-h urine protein quantification was 1.595 g (1.52, 2.37), 1.06 g (0.79, 1.63), and 0.315 g (0.27, 0.35), respectively (χ^2^ = 18.20, *p* < 0.001). Serum albumin showed an increase from 3.68 g/ dL (3,680 mg/dL, 3,530–3,770 mg/dL) to 4.075 g/dL (4,075 mg/dL, 3,910–4,200 mg/dL). eGFR remained stable during follow-up, changing from 108.3 mL·min^−1^·(1.73 m^2^)^−1^ (88.3, 112.3) to 113.95 mL·min^−1^·(1.73 m^2^)^−1^ (96.6, 119.7). All patients completed the 24-week follow-up.

**Conclusion:**

In this case series, add-on low-dose finerenone (10 mg/d) was associated with a significant reduction in residual proteinuria, an increase in serum albumin, stable renal function, and favorable safety in LN patients. These observational findings require validation in controlled trials.

## Introduction

Multiple prospective cohort studies confirm that residual proteinuria is an independent predictor of renal fibrosis progression, renal function decline, and adverse renal outcomes in LN, indicating incomplete control of renal inflammation, podocyte injury, and interstitial fibrosis. Thus, exploring safe and effective adjuvant therapies to eliminate residual proteinuria is clinically critical for improving long-term prognosis of LN.

Mineralocorticoid receptor (MR) overactivation plays a key role in chronic kidney disease-related renal injury. It aggravates glomerular filtration barrier damage, podocyte apoptosis, and tubulointerstitial fibrosis via pro-inflammatory, pro-oxidative stress, and pro-fibrotic pathways, driving proteinuria onset and persistence. Traditional steroidal MRAs (spironolactone, eplerenone) carry a high risk of hyperkalemia and are limited in patients with impaired renal function (1). Finerenone is a novel selective non-steroidal MRA with reduced electrolyte disorder risk (2). Large phase III randomized controlled trials (FIDELIO-DKD, FIGARO-DKD) confirm that finerenone significantly reduces proteinuria and renal composite endpoint events in diabetic kidney disease, and it is recommended in guidelines worldwide for this indication.

Growing evidence suggests the MR pathway contributes to renal injury in LN, and finerenone may have renal protective potential in LN. Recently, Sun et al. reported a pilot trial of finerenone 20 mg/d in 10 patients with histologically confirmed LN, demonstrating significant proteinuria reduction at 12 weeks (3). However, several clinically relevant questions remain unresolved: ① whether a lower dose of 10 mg/d is effective in LN—particularly in patients with preserved renal function (eGFR ≥ 60 mL/min/1.73 m^2^), where dose optimization remains unexplored; ② whether the proteinuria-lowering effect is sustained and quantifiable at both 12 and 24 weeks in the absence of concomitant SGLT2 inhibitors; and ③ whether detailed safety profiling, including serum potassium monitoring and eGFR trajectory, supports long-term use in this population. This retrospective case series addresses these gaps by evaluating add-on finerenone 10 mg/d over 24 weeks in 10 patients with clinically diagnosed LN and residual proteinuria on stable baseline immunosuppression and RASi, without SGLT2 inhibitor use.

## Methods

### Patients and study design

This study enrolled 10 patients with LN diagnosed at Weifang People’s Hospital, Shandong, China, from January 2023 to March 2026. All patients met the 2019 American College of Rheumatology revised SLE classification criteria. Patients had received stable low-dose glucocorticoids, immunosuppressants, and standard RASi for ≥ 6 months, with 24-h urine protein quantification > 0.5 g. All selected patients met the following criteria: serum potassium level < 4.8 mmol/L, estimated glomerular filtration rate (eGFR) ≥ 60 mL/min/1.73 m^2^. eGFR was calculated using the CKD-EPI 2009 equation based on serum creatinine. All enrolled patients fulfilled the 2019 ACR/EULAR classification criteria for systemic lupus erythematosus (SLE). No sex-based exclusion criteria were applied. The absence of male patients reflects consecutive real-world enrollment, consistent with the well-established female predominance of SLE and LN. Clinical diagnosis of lupus nephritis (LN) was established according to the renal involvement criteria of the 2019 ACR standard, defined as persistent proteinuria with 24-h urinary protein excretion >0.5 g after conventional baseline immunosuppressive and RAS inhibitor therapy. Owing to the retrospective nature of this clinical cohort, unified renal biopsy pathological data were not available for all participants. Therefore, in the present study, we prespecified the clinical assumption that SLE patients meeting the ACR-based clinical LN diagnosis and presenting with persistent residual proteinuria after standard foundational treatment were considered to have chronic LN complicated with residual proteinuria. Exclusion criteria: ① Patients who had received one course of methylprednisolone pulse therapy, or biologics, intravenous immunoglobulin, or plasma exchange treatment within 6 months; ② Patients with blood potassium > 5.0 mmol/L; ③ Patients with organ damage other than kidney damage, such as neurological damage, vasculitis, and myositis; ④ Patients with abnormal liver or kidney function; ⑤ Pregnant or lactating patients; ⑥ Patients with other important organ damage, such as heart disease, lung disease, malignant tumors, or accompanied by infection. This study was approved by the Ethics Committee of Weifang People’s Hospital, Shandong Province (approval number KYLL20260116-2), and all subjects gave informed consent. All patients maintained their original glucocorticoid, immunosuppressant, and RASi treatment regimens and were additionally treated with finerenone 10 mg once daily for 24 weeks. No patient was receiving sodium-glucose cotransporter 2 (SGLT2) inhibitors at baseline or during the 24-week follow-up period. The types and doses of basic treatment drugs were not adjusted during the follow-up period. The following indicators were measured and recorded at baseline, 12 weeks of treatment, and 24 weeks of treatment: ① Primary endpoint: 24-h urine protein quantification; ② Secondary endpoints: serum albumin, eGFR, serum potassium; ③ Safety endpoints: hyperkalemia (blood potassium > 5.0 mmol/L), acute kidney injury, hypotension, abnormal liver function, adverse drug reactions, and drug discontinuation events. Statistical analysis was performed using GraphPad Prism 9.0 software. Measurement data that did not follow a normal distribution were expressed as median (quartile, P25, P75); repeated measurement data were analyzed using the Friedman test; *p* < 0.05 was considered statistically significant. All continuous data are presented as median (25th percentile, 75th percentile).

## Results

### Baseline clinical data

All 10 enrolled patients were female, aged 31–61 years (median 43.5 years), with disease duration 4–7 years (median 5 years). Baseline treatments included: low-dose glucocorticoids (methylprednisolone 4–8 mg/d or prednisone 5–10 mg/d); mycophenolate mofetil (MMF) at 0.5–0.75 g twice daily (1000–1,500 mg/d) in 8 patients, or tacrolimus 1 mg twice daily in 2 patients; RASi (benazepril 10 mg once daily in 9 patients, telmisartan 40 mg once daily in 1 patient). All patients received hydroxychloroquine (200 mg/d) as part of baseline SLE management (Table 1).

**Table 1:** Changes in primary efficacy indicators before and after treatment.

Cases (*n*)	Sex	Age	Disease duration (years)	Immunosuppressant	RASi	Proteinuria (g/day) baseline	Proteinuria (g/day) 12 weeks	Proteinuria (g/day) 24 weeks	eGFR baseline	eGFR 24 weeks	Serum albumin baseline (mg/dL)	Serum albumin 24 weeks (mg/dL)
1	F	38	4	Methylprednisolone 4 mg qd, MMF 0.75 g bid (1,500 mg/d)	Benazepril 10 mg qd	1.52	0.89	0.17	112.3	114.9	3,670	3,890
2	F	58	5	Prednisone 10 mg qd, MMF 0.5 g bid (1,000 mg/d)	Benazepril 10 mg qd	2.74	1.67	0.41	88.3	95.3	3,720	4,200
3	F	39	6	Prednisone 10 mg qd, MMF 0.5 g bid (1,000 mg/d)	Benazepril 10 mg qd	2.37	1.63	0.3	112.1	116.3	3,690	4,190
4	F	55	5	Prednisone 7.5 mg qd, MMF 0.75 g bid (1,500 mg/d)	Benazepril 10 mg qd	1.75	0.79	0.27	61.2	68.4	3,460	4,330
5	F	38	6	Prednisone 5 mg qd, MMF 0.5 g bid (1,000 mg/d)	Benazepril 10 mg qd	1.27	0.35	0.26	108.9	110.9	3,530	4,010
6	F	33	6	Prednisone 5 mg qd, Tacrolimus 1 mg bid	Benazepril 10 mg qd	1.63	0.70	0.35	106.5	119.7	3,600	3,740
7	F	49	7	Prednisone 5 mg qd, MMF 0.75 g bid (1,500 mg/d)	Benazepril 10 mg qd	1.33	1.03	0.33	107.7	113.0	3,770	3,910
8	F	31	6	Methylprednisolone 8 mg qd, MMF 0.5 g bid (1,000 mg/d)	Benazepril 10 mg qd	1.56	1.09	0.28	118.6	124.7	3,950	430 0
9	F	61	5	Prednisone 5 mg qd, MMF 0.5 g bid (1,000 mg/d)	Telmisartan 40 mg qd	1.52	1.28	1.10	80.6	96.6	3,950	4,140
10	F	34	5	Methylprednisolone 8 mg qd, Tacrolimus 1 mg bid	Benazepril 10 mg qd	3.37	2.68	1.30	119.0	126.5	2,700	3,240
Median						1.60	1.06	0.315	108.3	113.95	3,680	4,075

MMF, mycophenolate mofetil; Serum albumin values are presented in mg/dL (1 mg/dL = 0.001 g/dL).

Baseline core parameters: 24-h urine protein quantification 1.595 g (1.52, 2.37); serum albumin 3.68 g/dL (3,680 mg/dL, 3,530–3,770 mg/dL).

### Efficacy outcomes

① 24-h urine protein quantification: showed a progressive decrease with the extension of treatment time, and was significantly lower at 12 weeks and 24 weeks compared to baseline, with highly statistically significant differences. ② Serum albumin: significantly increased after 24 weeks of treatment, indicating a reduction in proteinuria and improvement in nutritional status. ③ eGFR: There was no significant change before and after treatment, and renal function remained stable (Table 2).

**Table 2:** Statistical analysis (Friedman test was used, and *p* values were corrected by Bonferroni method).

Variable	Baseline	12 weeks	24 weeks	χ^2^	*P* value
Proteinuria (g/ day)	1.595 (1.52, 2.37)	1.06 (0.79, 1.63)*	0.315 (0.27, 0.35)*	18.20	<0.001

* *p* < 0.05 vs. baseline (Bonferroni-corrected for multiple comparisons).

### Safety results

No patient developed hyperkalemia (serum potassium >5.0 mmol/L). Serum potassium remained within the normal range throughout follow-up in all patients. No severe hyperkalemia (>5.5 mmol/L), acute kidney injury, hypotension, or liver function abnormalities were observed. No patients discontinued treatment due to drug adverse reactions, and all patients completed the 24-week follow-up.

## Discussion

The treatment goal of LN has shifted from inducing clinical remission via immunosuppression to achieving deeper organ protection and improved long-term prognosis. While glucocorticoid combined with cyclophosphamide or mycophenolate mofetil has elevated LN remission rates, residual proteinuria remains a common clinical challenge. These patients face elevated end-stage renal disease risk, as well as infections, myelosuppression, and metabolic disorders from long-term high-dose glucocorticoids and immunosuppressants.

Recent studies show MR overactivation exacerbates glomerular basement membrane damage and interstitial fibrosis via pro-inflammatory and pro-fibrotic pathways, closely linked to LN chronic progression. Finerenone, a third-generation non-steroidal MRA, has higher receptor selectivity and balanced tissue distribution than spironolactone. Large outcome trials in type 2 diabetes-related chronic kidney disease confirm finerenone reduces proteinuria and delays eGFR decline, but prospective evidence in immune-mediated glomerular diseases remains limited (4). Emerging evidence supports the expansion of non-steroidal MRAs beyond diabetic kidney disease. A recent review highlighted the rationale for nsMRA use in non-diabetic CKD and glomerular diseases (5). Notably, the phase 3 FIND-CKD trial has recently demonstrated that finerenone significantly reduces kidney function decline in patients with non-diabetic CKD (6), broadening the therapeutic landscape for non-diabetic renal conditions. However, FIND-CKD excluded patients with active autoimmune glomerular diseases such as LN; therefore, the efficacy and safety of finerenone in immune-mediated proteinuria remain to be specifically addressed. In this context, our observational data contribute to the preliminary real-world evidence supporting the translational potential of finerenone from diabetic to non-diabetic immune-mediated kidney disease.

Our findings complement the recently published pilot trial by Sun et al. (3), who evaluated finerenone 20 mg/d in histologically confirmed LN. While both studies observed substantial proteinuria reduction, our study differs in three aspects: ① we employed a lower dose (10 mg/d) in a SGLT2 inhibitor-free cohort, allowing a less confounded assessment of finerenone’s specific effect; ② we provide formal repeated-measures analysis at both 12 and 24 weeks; and ③ our detailed safety profiling, including serum potassium trajectory, supports the tolerability of low-dose finerenone in patients with preserved renal function. In our cohort, the enrolled patients had received ≥ 6 months of standardized glucocorticoid and immunosuppressant therapy, with long disease duration, no active inflammation or extra-renal damage, and persistent non-remitting proteinuria. After 24 weeks of add-on finerenone 10 mg/d, median urine protein showed a decrease from 1.595 g to 0.315 g (80.2% reduction), with concurrent increases in serum albumin. These observations suggest that low-dose finerenone (10 mg/d) may be associated with significant proteinuria reduction. Meanwhile, eGFR remained stable, indicating no adverse renal impact, and serum potassium showed no significant elevation, supporting this strategy as a new add-on option for refractory LN. The magnitude of this reduction warrants cautious interpretation. While the 80% proteinuria reduction numerically exceeds the ~30–40% observed in FIDELIO-DKD and FIGARO-DKD, and could reflect differential pathophysiology—autoimmune glomerular injury in LN may be more responsive to MR blockade than diabetic microangiopathy, with background immunosuppression leaving MR-driven mechanisms as the dominant residual driver—we emphasize that the small sample size, absence of a control group, and potential for regression to the mean render this observation unreliable. These findings are hypothesis-generating only and require confirmation in prospective randomized trials.

MR activation contributes to glomerular injury, vascular disease, and tubulointerstitial fibrosis (7). Aldosterone-mediated MR overactivation upregulates pro-inflammatory cytokines, reactive oxygen species, and connective tissue growth factor, promoting renal inflammation and fibrosis. MR antagonists exert renal protection via multiple mechanisms, and preclinical studies confirm MR blockade reduces proteinuria, alleviates renal injury, and prevents glomerulosclerosis (8–10). In LN patients, MR signaling promotes inflammatory factor release via NF-κB activation, inducing podocyte apoptosis and mesangial matrix proliferation. Although ACEI/ARB blocks angiotensin II, long-term use may cause “aldosterone breakthrough” and MR reactivation. The proteinuria-lowering and cardiovascular protective effects of finerenone demonstrated in diabetic kidney disease may be translatable to LN patients (11). Classic MRAs are limited by hyperkalemia in chronic kidney disease. In this study, no serum potassium > 5.5 mmol/L or treatment discontinuation occurred, consistent with the FIDELITY pooled analysis (hyperkalemia-related hospitalization rate < 1%). Finerenone’s non-steroidal structure confers negligible affinity for sex hormone receptors, with no gynecomastia or menstrual disorders observed—an important advantage for the predominantly female LN population. Notably, median baseline eGFR was > 60 mL·min^−1^·(1.73 m^2^)^−1^; efficacy and safety in severe chronic kidney disease (eGFR <30 mL·min^−1^·(1.73 m^2^)^−1^) require further validation.

This study has important limitations. As a single-center retrospective case series without a concurrent control group, we are unable to delineate the individual contribution of finerenone from the concurrent effects of ongoing background immunosuppression, spontaneous disease fluctuation, or regression to the mean. These findings therefore represent preliminary observations that are intended to be hypothesis-generating and warrant confirmation in prospective controlled trials. Additional considerations include the small sample size, the 24-week follow-up period (which precludes assessment of long-term renal protection). All enrolled patients were female, reflecting the epidemiology of SLE rather than deliberate selection; however, this limits generalizability to male patients.

SGLT2 inhibitors have demonstrated renoprotective effects in diabetic kidney disease and are increasingly recognized for their potential in LN. In this study, the absence of SGLT2 inhibitors allows a less confounded assessment of finerenone’s specific effect on residual proteinuria. We believe that LN patients, particularly those with metabolic comorbidities, may benefit from SGLT2 inhibition. Whether finerenone retains its proteinuria-lowering effect when combined with SGLT2 inhibitors—and whether this combination provides additive cardiorenal protection in LN—should be evaluated in future trials. The pilot trial by Sun et al., in which 40% of patients received SGLT2 inhibitors, provides preliminary support for the tolerability of such combination therapy (3). However, the results of this study truly reflect the application value of finerenone in clinical practice, especially confirming that the low dose of 10 mg/d has a good benefit–risk ratio in LN patients, providing a basis for subsequent prospective, randomized controlled studies.

## Conclusion

In this retrospective case series, add-on low-dose finerenone (10 mg/d) was associated with a reduction in 24-h urine protein quantification over 24 weeks in patients with LN and residual proteinuria despite standard immunosuppression, alongside stable renal function and favorable short-term safety profiles. These observational findings, while encouraging, represent preliminary evidence that warrants confirmation in prospective controlled studies to further elucidate the relationship between finerenone and proteinuria dynamics in this population.

## Data Availability

The raw data supporting the conclusions of this article will be made available by the authors, without undue reservation.
